# Agranulocytosis Following COVID-19 Recovery

**DOI:** 10.7759/cureus.9463

**Published:** 2020-07-29

**Authors:** Forat Lutfi, Amin Benyounes, Naveed Farrukh, Jacqueline Bork, Vu Duong

**Affiliations:** 1 Oncology, University of Maryland Medical Center, Baltimore, USA; 2 Hematology and Oncology, University of Maryland Medical Center, Baltimore, USA; 3 Internal Medicine, University of Maryland Medical Center, Baltimore, USA; 4 Infectious Disease, University of Maryland Medical Center, Baltimore, USA; 5 Hematology and Medical Oncology, University of Maryland Medical Center, Baltimore, USA

**Keywords:** agranulocytosis, neutropenia, coronavirus, covid-19

## Abstract

Clinicians have continued to report on the clinical behavior and characteristics of patients with coronavirus disease 2019 (COVID-19) as our knowledge of the virus continues to mature. Herein, we report the case of a 39-year-old male with multiple comorbidities who became critically ill with COVID-19 infection, requiring mechanical ventilation and vasopressors, and then developed agranulocytosis following clinical improvement and resolution of symptoms of COVID infection. The period of agranulocytosis coincided with the development of thrombocytosis, and following resolution of agranulocytosis, the platelet count also normalized, suggesting a possible related mechanism. Interestingly, the patient was treated with TBO-filgrastim 480 mcg daily with a rapid reconstitution of neutrophils. While the mechanism of agranulocytosis remains unknown, we report, to our knowledge, the first known case of agranulocytosis following COVID-19 infection and its successful treatment with granulocyte colony-stimulating factor.

## Introduction

Since its emergence in late 2019 and now being declared a pandemic by the World Health Organization (WHO), the severe acute respiratory syndrome novel coronavirus 2 (SARS-nCoV-2) has infected over one million individuals in the United States and has a highly variable clinical presentation [1]. Hematologic laboratory abnormalities in infected patients have been reported, including lymphopenia and neutrophilia, and are directly correlated with clinical severity [2]. We present a case of a young man with agranulocytosis following coronavirus disease 2019 (COVID-19) infection responding to granulocyte colony-stimulating factor (G-CSF), and, to our knowledge, this has not been reported in the literature.

## Case presentation

A 39-year-old African American male with a previous history of reduced ejection fraction heart failure, hypertension, insulin-dependent type 2 diabetes mellitus, gout, morbid obesity, and chronic kidney disease stage III presented with progressive fever, dyspnea, and non-productive cough of several days’ duration.

**Figure 1:**
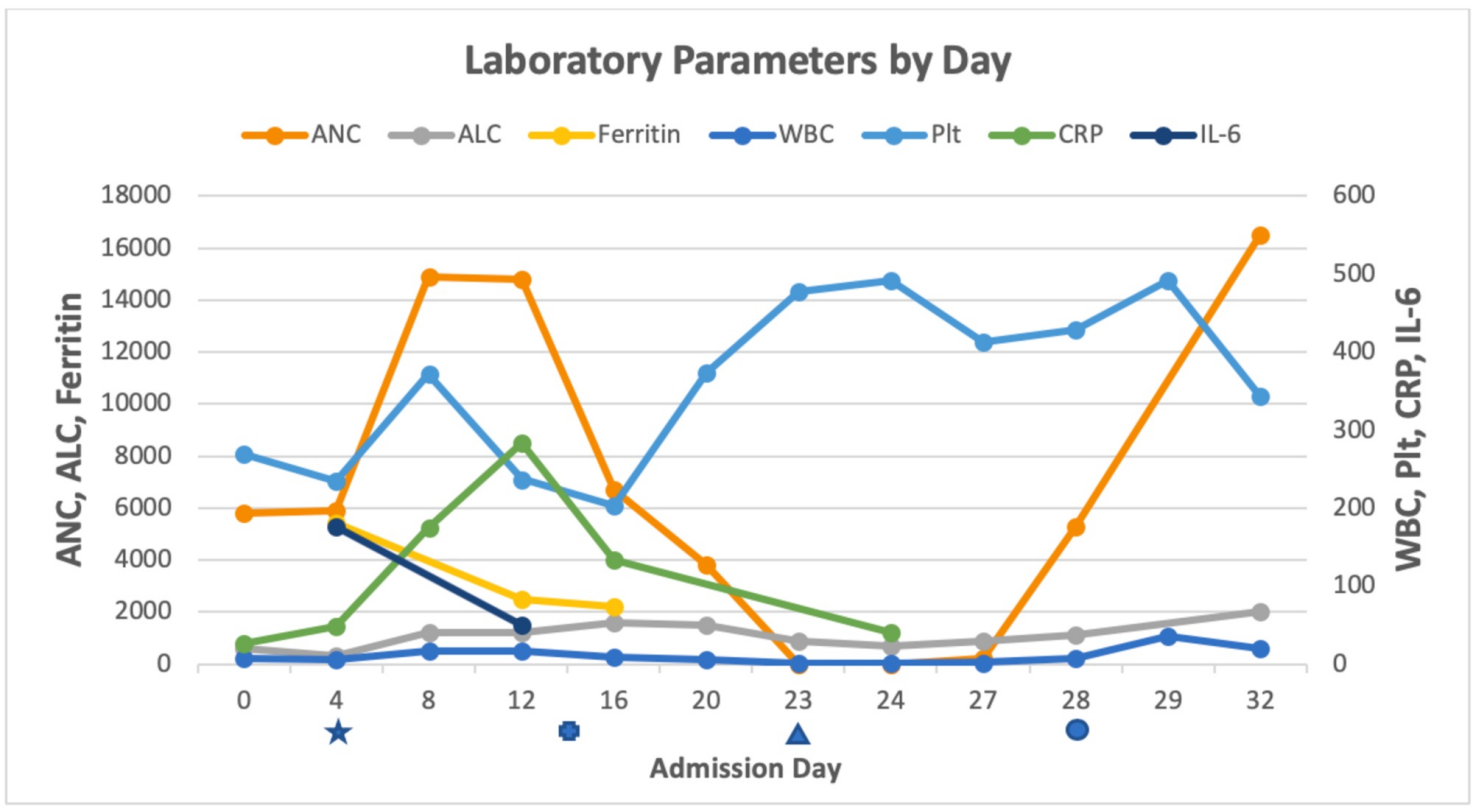
Clinical and Laboratory Timeline The star denotes intubation, cross denotes extubation, triangle denotes start of granulocyte colony-stimulating factor (G-CSF), and circle denotes end of G-CSF. ANC, absolute neutrophil count; ALC, absolute lymphocyte count; WBC, white blood cell count; Hgb, hemoglobin; Plt, platelets; CRP, C-reactive protein; IL-6, interleukin-6

At the time of admission, he had a normal white blood cell count (WBC) of 6,900/mcL with an absolute neutrophil count (ANC) of 5,800/mcL, lymphopenia with an absolute lymphocyte count (ALC) of 600/mcL, hemoglobin (Hgb) of 15.8 g/dL, platelets of 276,000/mcL, and elevated C-reactive protein (CRP) of 41.2 mg/dL. COVID-19 infection was confirmed by polymerase chain reaction (PCR). He rapidly developed cardiogenic shock, renal failure, and respiratory failure requiring vasopressors, renal replacement therapy, and mechanical ventilation on day 4. He received hydroxychloroquine on hospital days 3-7 and azithromycin on days 6-7 for COVID-19 infection as an off-label, non-clinical trial treatment. His CRP peaked at 286.7 mg/dL on day 12, which also correlated with the peak in the severity of his clinical illness. On that day, the ANC was 14,800/mcL, ALC had normalized to 1,200/mcL, Hgb had declined to 7.5 g/dL, and platelet count was 237,000/mcL (Figure 1). By day 14, his respiratory status had improved and he was extubated. Although he improved clinically, remained afebrile with negative blood cultures, and CRP was decreasing, he continued to have a positive COVID-19 PCR assay. He then developed rapidly worsening neutropenia and thrombocytosis with ANC decreasing to 0 and platelets increasing to 478,000/mcL by hospital day 23. The peripheral smear showed no neutrophils and few atypical lymphocytes. Medications from the time of admission were reviewed, and no potential culprits were identified (Table 1). The patient was initiated on TBO-filgrastim 480 mcg daily starting on day 23 with rapid reconstitution of neutrophils, reaching an ANC of 5,300/mcL by day 28 when growth factor support was stopped. His ANC remained greater than 1,000/mcL for the remainder of the hospitalization. The thrombocytosis also resolved with platelets reaching normal values by day 32 (343,000/mcL).

**Table 1:** Medications Administered from Day 0 to Day 23

Medication	Day(s) administered	Neutropenia described
Heparin	0-23	Yes
Vancomycin	1-20	Yes
Piperacillin/tazobactam	1-9	Yes
Hydroxychloroquine	3-7	Yes
Fentanyl	4-7	
Pantoprazole	3-20	
Ipratropium	5-14	
Albuterol	5-14	
Azithromycin	6-7	Yes
Quetiapine	11-20	
Amiodarone	16-20	Yes
Meropenem	12-18	Yes
Metoprolol	16-23	
Digoxin	17-22	
Midodrine	18	
Dobutamine	3	
Propofol	5-7	
Vasopressin	7-11	
Acetaminophen	5-13	
Sevelamer	1-23	
Atorvastatin	2-6	
Midazolam	8-11	
Dexmedetomidine	6-16	
Norepinephrine	6-7, 11-13, 16, 20	
Phenylephrine	7-9, 16	
Ceftriaxone	16	Yes
Haloperidol	14-15	Yes
Guaifenesin	13-19	
Insulin NovoLog^®^	1-20	
Melatonin	14-23	
Sennosides	9-23	
Polyethylene glycol	9-20	
Diphenhydramine	20-23	

## Discussion

In the midst of a new pandemic with COVID-19 and its heterogenous clinical presentation, clinicians are striving to learn the symptomatology, laboratory, and diagnostic characteristics to determine effective therapeutic approaches for patients. As observed in this patient, lymphopenia, neutrophilia, and elevated CRP initially correlated with the severity of his illness with COVID-19; however, to our knowledge, the development of agranulocytosis and thrombocytosis in the recovery phase, following resolution of symptoms, has yet to be described. Interestingly, a mechanism of SARS-nCoV-2 entering T-lymphocytes by viral spike protein binding to CD147 expressed on the surface of T-lymphocytes has been described, although its effect on lymphopenia remains unknown [3]. CD147 is also expressed on neutrophils and platelets, but the impact of SARS-nCoV-2 binding to CD147 on their function and survival remains unknown [4]. Bone marrow suppression and peripheral destruction leading to neutropenia have been well described in other viral infections including HIV, cytomegalovirus, Epstein-Barr virus, viral hepatitis, and influenza, although it has yet to be described in SARS-nCoV-2, and most commonly coincides with clinical severity [5]. Other potential etiologies of agranulocytosis, namely medications and other concurrent infections, were less likely given the time course of their administration and the subsequent development of agranulocytosis. We note that this patient was treated with hydroxychloroquine off-label and off-trial early in the pandemic, when its use was more common, and that hydroxychloroquine has rarely been reported to cause agranulocytosis. However, it was administered for only five days, and given that the last dose was more than two weeks prior to the development of agranulocytosis, it seems to be an unlikely contributing factor in this case [6]. Additionally, in hydroxychloroquine-induced agranulocytosis, it has been reported to occur after months, not days, of use [6-7]. Furthermore, while severe sepsis and critical illness are known to cause neutropenia and agranulocytosis, it is not typically this profound and occurs when illness is most severe, not following recovery. We acknowledge that a bone marrow biopsy and anti-neutrophil antibody testing may have been clinically useful; however, given rapid normalization of neutrophil count with G-CSF, they were not obtained.

## Conclusions

The co-development of agranulocytosis with thrombocytosis followed by rapid resolution with growth factor support suggests a temporal and reactive phenomenon in the setting of COVID-19 infection. Although agranulocytosis may be a rare manifestation following COVID-19 infection, clinicians should be aware of this phenomenon, as it may have implications on patient management and ultimately patient outcomes during this time period. Furthermore, G-CSF may be used effectively to treat agranulocytosis following infection with COVID-19.
